# Influence of Clinical Experience on Learning the Focused Assessment With Sonography for Trauma (FAST) Protocol: A Pilot Study

**DOI:** 10.7759/cureus.99294

**Published:** 2025-12-15

**Authors:** Cristiano M Quintão, Gabriela P Domingues, Antonio Toledo

**Affiliations:** 1 Health Department, Prefeitura Municipal de Conceição do Mato Dentro, Conceição do Mato Dentro, BRA; 2 Internal Medicine Department, Universidade Professor Edson Antônio Velano, Belo Horizonte, BRA; 3 Internal Medicine Department, Faculdade Ciências Médicas de Minas Gerais, Belo Horizonte, BRA

**Keywords:** bedside diagnosis, focused assessment with sonography for trauma (fast), medical education, point-of-care ultrasound (pocus), ultrasonography

## Abstract

Background

Point-of-care ultrasound (POCUS), and specifically the Focused Assessment with Sonography for Trauma (FAST) protocol, is widely used to expedite the assessment of trauma patients at the bedside, but optimal strategies for teaching FAST and the role of prior clinical experience in learning remain unclear. Brief, structured training programs are increasingly proposed for undergraduate and early postgraduate curricula, yet evidence comparing learners with different clinical backgrounds is limited.

Objective

This study aimed to compare the performance of physicians and final-year medical students without prior ultrasound experience in learning the FAST protocol after structured training.

Materials and methods

This is a quasi-experimental, non-blinded pilot study. Participants were non-imaging physicians and final-year medical students. All received a standardized four-hour theoretical-practical FAST training and completed pre- and post-written tests. Two weeks later, the participants underwent practical assessments on human models and an ultrasound simulator. Scores were converted to percentages for comparability. Normality was assessed with the Shapiro-Wilk test. Descriptive analyses were done. Comparative analysis included between-group comparisons and pre- and post-test paired comparisons, including effect size. Parametric or non-parametric tests were used as appropriate. Two-sided tests were used with α = 0.05. Effect sizes and p-values were computed in JASP v0.19.3 (University of Amsterdam, Netherlands).

Results

Forty volunteers participated (physicians n = 19; final-year medical students n = 21). Physicians scored higher than students on both pre- and post-training written tests (pre-test total: 53.6% vs. 39.0%, p = 0.004; post-test total: 76.1% vs. 60.6%, p < 0.001). Paired analyses showed significant improvements across all written components for both groups, with very large paired effect sizes (rank-biserial correlation (RBC) ranged from 0.77 to 1.00); the largest absolute gain was in image identification among students (+29.5 percentage points, RBC = 0.86) and in theoretical assessment among physicians (+28.1 points, RBC = 1.00). In Phase 2 practical assessments (n = 37), no statistically significant differences were found between groups in overall or FAST-specific scores, but students completed tasks substantially faster (mean time ≈5 min vs. ≈10 min for physicians; p < 0.001). Diagnostic accuracy in the simulated cases was high in both groups; only one physician made an incorrect diagnosis (p = 0.245). Overall, brief structured FAST training produced large within-subject gains in knowledge and skills across both learner groups.

Conclusions

Brief structured FAST training produced substantial gains in knowledge and skills for both groups. Comparable practical performance between physicians and students suggests that procedural competence can be effectively acquired through structured training regardless of prior clinical experience, supporting early integration of ultrasonography training into undergraduate curricula.

## Introduction

Point-of-care ultrasound (POCUS) is a non-invasive examination tool that guides immediate clinical decisions. In many situations, it can obviate other imaging tests, avoiding unnecessary transfers, reducing imaging service overload, and limiting ionizing radiation exposure [1,2]. With technological advances and the dissemination of portable devices, POCUS has expanded into multiple contexts, including emergency services and remote areas [3]. In these settings, POCUS becomes a strategic tool by enabling rapid, real-time evaluations in potentially critical patients [1,4].

Among its applications, the FAST protocol stands out for rapidly identifying life-threatening conditions such as cardiac tamponade and internal hemorrhage, supporting early clinical decisions with high specificity [5]. This is particularly important because approximately 10% of victims of high-energy trauma present with nonspecific clinical signs that are not always detectable by physical examination alone [6]. POCUS use can significantly reduce the interval between admission, diagnosis, and treatment initiation, improving outcomes and survival [7], and may also decrease the need for complementary tests and length of stay, underscoring its value in emergency care, internal medicine, and resource-limited settings [8].

Despite these benefits, accuracy is directly related to operator experience, reinforcing the importance of structured training and supervised practice [5]. When performed by adequately trained physicians, FAST accuracy can exceed 85%, making it a reliable and accessible resource in the assessment of polytrauma patients [9].

Medical educators increasingly advocate for early inclusion of POCUS in curricula, with evidence that learning this tool strengthens anatomical understanding, imaging proficiency, and real-time decision-making [10]. This approach also promotes integration between theory and practice, positively affecting knowledge retention and clinical application [11,12]. Experiences in countries such as the United States, Canada, and Germany show that structured undergraduate POCUS teaching improves academic performance and clinical skills [13,14]. However, challenges remain, including a lack of curricular standardization, limited time for practice, and infrastructure constraints [11,15,16].

Medical training involves not only technical mastery but also the development of professional identity through clinical experience. Exposure to real patients contributes to clinical reasoning, decision-making under pressure, and judicious interpretation of diagnostic tests [17,18]. Because POCUS is operator-dependent, prior clinical experience may influence how physicians and students learn the technique and interpret results, even when both groups lack ultrasound training. It remains unclear whether general clinical experience confers advantages in acquiring basic POCUS skills among novice learners. We therefore compared the performance of physicians and final-year medical students with no prior ultrasonography experience in performing the FAST protocol in a simulated environment after standardized theoretical-practical training.

## Materials and methods

This was a quasi-experimental, non-blinded pilot study with parallel groups conducted in two phases. Phase 1 consisted of a standardized theoretical-practical training on the FAST protocol, and Phase 2 consisted of a practical assessment two weeks later. Random allocation was not feasible due to operational and ethical constraints. Operationally, the physician group comprised almost all eligible practitioners from the partnered city where the project was implemented. Ethically, randomizing students within their own institution to receive or withhold educational content raised concerns about educational equity and potential coercion.

Participants

The study was conducted in two locations approximately 170 km apart: a private medical school in Belo Horizonte, Minas Gerais, Brazil, and a small city (Conceição do Mato Dentro, Minas Gerais) where the project was implemented as part of an educational partnership with the local health department. This geographic separation, combined with independent on-site training and assessment at each location, minimized the risk of cross-contamination between groups.

The study population consisted of final-year medical students from the medical school and physicians who had graduated within the past 10 years and were working in primary care in the partnered city. Inclusion criteria were age ≥18 years and signed informed consent. Exclusion criteria were specialization in ultrasonography or imaging, or prior training in POCUS or FAST. Recruitment occurred via in-person announcements (classes/meetings), social media, and snowball sampling. 

A convenience sample targeted 20 participants per group. This sample size was chosen pragmatically based on logistical constraints and conventional recommendations for pilot studies in medical education, which typically include 15-25 participants per group to estimate effect sizes [19]. 

Training and assessment

For Phase 1, a standardized theoretical-practical training on the FAST protocol was developed based on the protocol by Monti and Perreault for novice military medics [20]. The training lasted four hours and was delivered by the same two physician instructors to both groups. The first two hours were devoted to theoretical instruction (lectures and image review) and the remaining two hours to supervised hands-on practice. The practical component included hands-on ultrasound scanning on a lean human model to learn probe handling and image acquisition, followed by training on an ultrasound simulator (SonoSim®, United States) with pathological cases for practice in recognizing abnormal findings. 

Two weeks after training, participants were individually assessed. Although they were aware of the assessment, they were not informed of its specific content. All participants waited in a separate room before testing and were called individually, with no contact between those who had completed the exam and those awaiting evaluation. These procedures ensured standardized conditions and prevented intragroup contamination. Each participant first performed the FAST protocol on a human model and was evaluated by the FAST Image Acquisition Checklist (FIAC). The participant then completed the FAST-Specific Assessment (FSA) using the SonoSim® simulator, each supervised by different instructors.

Instruments

In Phase 1, after the consent term signature, the participants completed a sociodemographic questionnaire. The same written test was administered before and after training. It comprised six multiple-choice questions addressing FAST fundamentals, clinical indications, accuracy, and clinical decision-making. Additionally, five image-identification items required recognition of the standard FAST windows using normal ultrasonographic images. Each correct response scored one point, with a maximum score of 11 points.

In Phase 2, the assessment instruments were adapted from previously published and validated tools [20,21]. The practical evaluation used two instruments. The first one (FIAC) was a checklist based on the Quality of Ultrasound Imaging and Competence (QUICk) Score developed by Ziesmann et al. for objective assessment of image acquisition during FAST examinations [21]. The second instrument (FSA) evaluated the FAST performance based on the model proposed by Monti and Perreault [20]. The instruments were adapted for local use without formal validation into Portuguese, as they served as structured observation guides for trained evaluators rather than participant-facing tools. Each station was scored by a single evaluator using the corresponding checklist for all participants, ensuring intra-rater reliability.

FIAC consisted of 24 items rated on a five-point Likert scale (1 = very poor to 5 = very good; maximum score = 120 points). This checklist assessed probe handling, image acquisition, and identification of key anatomical structures, and was applied to a human model.

FSA included 14 dichotomous items (1 = correct, 0 = incorrect; maximum score = 14 points) and one open question about exam normality. It evaluated whether participants correctly assessed each FAST window and identified the relevant anatomical structures, using an ultrasound simulator (SonoSim®, United States). In addition, participants were required to indicate whether any abnormality was present and to identify the specific finding. The simulator was set to show a case of blunt abdominal trauma with splenic rupture and intraperitoneal free fluid. Execution time was measured in FIAC and FSA.

Higher scores indicated better performance in the three instruments.

Data analysis

The main independent variable was group (physician vs. student). The primary outcome was practical performance (assessment scores); the secondary outcome was the post-training written test score. All scores were converted to percentage values for comparability. Descriptive statistics were produced, and normality was assessed with the Shapiro-Wilk test. Between-group comparisons used chi-square for categorical variables, and Student’s t or Mann-Whitney U tests for continuous variables, as appropriate. Within-subject (pre-post) comparisons used paired t or Wilcoxon signed-rank tests, as appropriate. Effect sizes were reported as rank-biserial correlation for nonparametric tests and standardized mean differences (Hedges’ g) for parametric tests. A two-tailed α = 0.05 was used for all hypothesis tests. Analyses were performed in JASP v0.19.3 (University of Amsterdam, Netherlands).

Participants who did not complete the first phase were excluded from the study, and those who did not complete the final assessment were excluded from the Phase 2 analysis. No imputation was performed for missing data, and all analyses were conducted on complete cases.

Ethics

The study was approved by the Institutional Human Research Ethics Board (registration 75791923.0.0000.5143; approval number 6.584.677) and was conducted in accordance with the Declaration of Helsinki. All participants provided written informed consent prior to inclusion.

## Results

**Figure 1:**
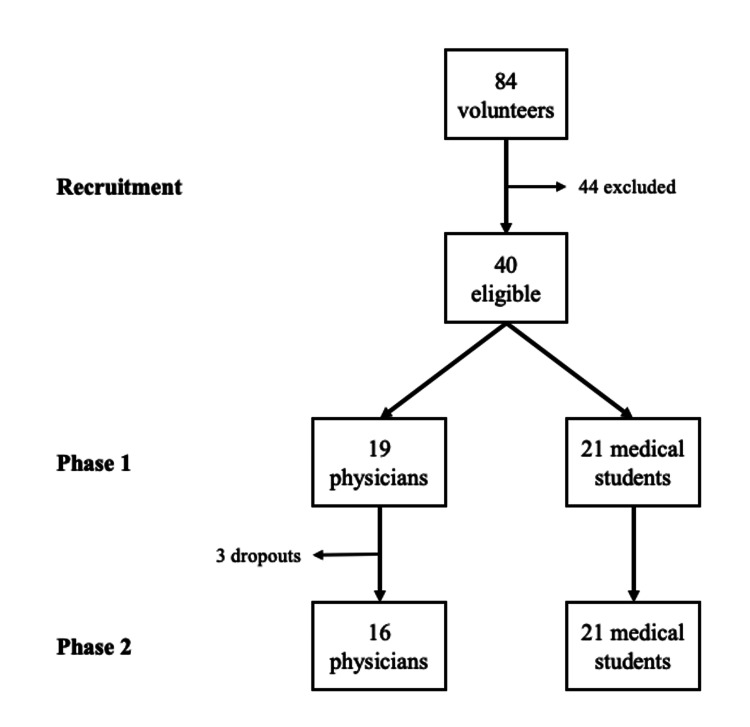
Flow diagram of participants through each phase of the study

Between June and September 2024, 84 potential volunteers were screened; 40 met eligibility criteria and provided informed consent (19 physicians, 21 medical students). Physician training occurred on June 15 and assessment on June 29, 2024. Students were trained on August 31 and assessed on September 14, 2024. Three physicians did not attend the final assessment and were excluded from Phase 2 analysis. Thus, the final samples were 19 physicians and 21 students at Phase 1 and 16 physicians and 21 students at Phase 2 (Figure 1).

Most physicians were male (68.4%) and worked as general practitioners (without a medical specialty). The mean time since graduation was 5.0 years (± 3.1; range <1-10 years), and the median was 4.0 years (interquartile range 2.5-8.0 years). Among medical students, 52.4% were male (Table 1).

**Table 1:** Comparative analysis of demographic and professional characteristics of the 40 volunteers

Variable		Physicians (n = 19)		Medical students (n = 21)	
		n	%	n	%
Sex	Female	6	31.6	10	47.6
	Male	13	68.4	11	52.4
Specialty (physicians only)	General practitioner	12	63.2	---	---
	Pediatrics	3	15.8	---	---
	Internal medicine	2	10.5	---	---
	Obstetrics-gynecology	2	10.5	---	---

Phase 1: written assessments

In the pre-test, physicians scored higher than students in image identification and in total scores (p < 0.05), while theoretical scores did not differ significantly (Table 2). The mean overall performance in both groups was below 60%. After training, physicians achieved higher scores across all components (p < 0.05). Theoretical performance exceeded image-identification performance in both tests. 

**Table 2:** Written assessments performance by group (n = 40) SD: standard deviation; * Mann-Whitney U test (nonparametric)

Variable	Physicians (n = 19) Mean (SD)	Medical students (n = 21) Mean (SD)	U	p*
Pre-test (theory)	56.1 (17.8)	51.6 (16.6)	225.5	0.472
Pre-test (image)	50.5 (18.1)	23.8 (24.2)	314.5	0.001
Pre-test (total)	53.6 (13.1)	39.0 (14.4)	305.0	0.004
Post-test (theory)	84.2 (13.0)	66.7 (13.9)	320.0	<0.001
Post-test (image)	66.3 (13.4)	53.3 (17.1)	296.5	0.004
Post-test (total)	76.1 (9.2)	60.6 (11.6)	337.0	<0.001

Table 3 summarizes the effect size of the training by assessment component. Both groups showed significant improvement in all components after training, with very large effect sizes (Rank-Biserial Correlation ≥ 0.70). The greatest gain among physicians occurred in the theoretical component (+28.1 percentage points), while among students it was in image identification (+29.5 points).

**Table 3:** Paired analysis of the training effect on pre–post written assessments (n = 40) *Wilcoxon signed-rank test (paired), **Rank-biserial correlation (post > pre-test)

Group	Component	Pre-test	Post-test	Difference (pp)	Z	p*	Effect size**
Physicians	Theoretical assessment	56.1	84.2	28.1	-3.62	<0.001	1.00
(n = 19)	Image identification	50.5	66.3	15.8	-3.62	<0.001	1.00
	Total performance	53.6	76.1	22.5	-2.35	0.018	0.77
Medical students	Theoretical assessment	51.6	66.7	15.1	-3.72	<0.001	1.00
(n = 21)	Image identification	23.8	53.3	29.5	-3.02	0.003	0.86
	Total performance	39.0	60.6	21.6	-3.48	<0.001	0.96

Phase 2: practical assessments

No statistically significant differences between groups were found in FIAC or FSA scores (p > 0.05) (Table 4). However, students completed the practical assessments in approximately half the time required by physicians (p < 0.05). Diagnostic accuracy in FSA was high in both groups; only one physician made an incorrect diagnosis.

**Table 4:** Practical assessments performance by group (n = 37) SD: standard deviation; * Mann-Whitney U test (nonparametric)

Variable	Physicians (n = 16) Mean (SD)	Medical students (n = 21) Mean (SD)	U	p*
FAST Image Acquisition Checklist (FIAC)	83.9 (11.5)	89.5 (9.5)	124.0	0.172
Time (FIAC), min	9.8 (3.1)	5.2 (1.2)	301.0	<0.001
FAST-Specific Assessment (FSA)	84.0 (5.4)	80.9 (5.4)	223.5	0.09
Time (FSA), min	10.4 (2.6)	5.9 (1.2)	320.5	<0.001

**Figure 2:**
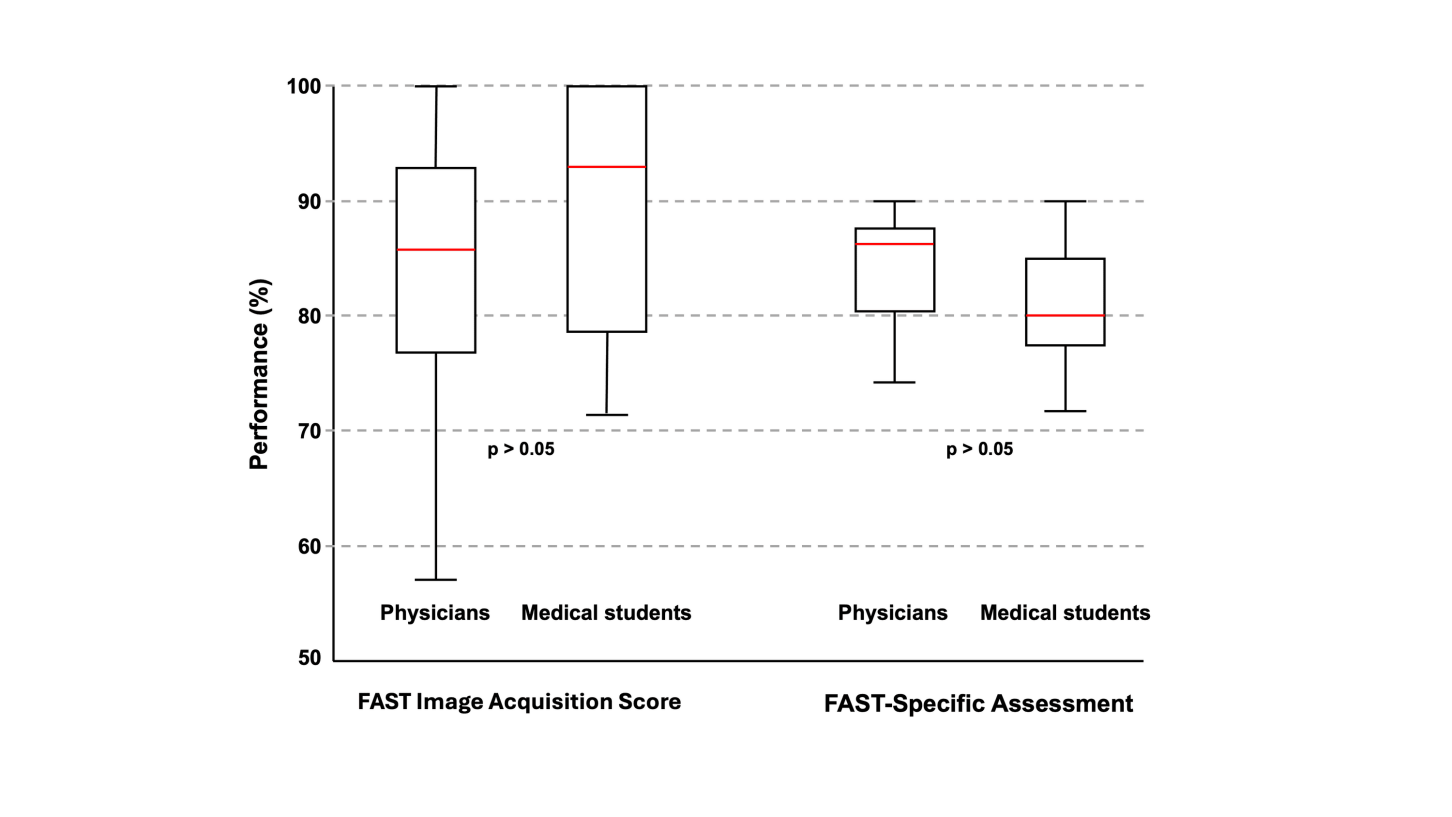
Boxplots comparing performance on the general practical assessment and the FAST-specific assessment for the 37 volunteers (physicians, n = 16; medical students, n = 21) FAST: Focused Assessment With Sonography for Trauma

Score distributions were similar across groups, with most participants achieving values above 75% on both practical stations. However, performance variability was greater among students, particularly in the general ultrasound assessment (Figure 2).

## Discussion

This study compared the performance of physicians and medical students without prior ultrasonography training in the FAST protocol. The training was effective in both groups, with significant post-test gains and very large effect sizes across all components. Physicians' higher post-training scores likely reflect greater clinical experience and more frequent exposure to diagnostic imaging [14,15,22,23]. By contrast, practical performance did not differ significantly between groups, likely reflecting both groups' limited hands-on experience with POCUS. This finding supports the notion that structured and supervised training can be effective even for novice learners [23].

Despite similar final performance, students completed the examinations in approximately half the time of physicians. This finding requires careful interpretation. Clinical experience may make physicians more thorough and cautious, as suggested by previous studies [18, 24]. More experienced clinicians commonly perform systematic scanning of multiple anatomical planes, thereby prolonging examination time, even in simulated settings [25]. In addition, medical students may benefit from more recent anatomical knowledge, since early-stage ultrasound training relies primarily on recognition of anatomical structures [26, 27]. Furthermore, their familiarity with simulated environments and practical assessments may also promote a rapid, task-oriented execution, sometimes at the expense of thoroughness [25].

Shorter execution time should not be mistaken for real-world clinical efficiency. In emergency settings, experienced operators typically perform the FAST examination within two to four minutes [28], as a result of extensive exposure and motor pattern consolidation. In contrast, the present study involved novice participants who received only four hours of training, including two hours of hands-on practice. Therefore, the observed differences in execution time likely reflect task familiarity and test-taking behavior rather than true clinical proficiency. Physicians may also have approached the task with a stronger sense of clinical responsibility, performing the examination more cautiously and systematically, which naturally prolonged execution time. Importantly, diagnostic accuracy remained high and comparable between groups, underscoring that reduced time did not compromise result quality.

The influence of the simulated environment should also be acknowledged. While simulation provides a safe and standardized setting for skill acquisition, it may inadvertently encourage a task-oriented approach focused on speed rather than diagnostic accuracy. Even among final-year students, the simulated practice can be perceived as a game-like exercise rather than a representation of genuine clinical responsibility. Therefore, future educational interventions must emphasize the primacy of diagnostic accuracy over execution time and explicitly integrate clinical reasoning and patient safety principles into simulation-based ultrasound training.

The optimal timing for teaching point-of-care ultrasound remains debated. Some authors argue that residency is the most appropriate period, given the intensive contact with real clinical situations [5]. However, the results of the present study indicate that FAST can also be taught during undergraduate training, provided as part of a structured program. This view is reinforced by Kondrashova and Coleman, who emphasize the effectiveness of curricula adapted to different levels of training [26]. It should also be noted that students display strong motivation to learn ultrasonography, driven both by the perceived professional utility of the technique and by its practical nature [26,27,29,30]. 

This study has some limitations. The sample size was relatively small. However, it was adequate for a pilot study in medical education, designed primarily to test feasibility and estimate effect sizes rather than to produce definitive conclusions. In addition, participants were not randomized. Notably, randomization was not feasible due to the operational and ethical constraints, but the use of standardized content and the same instructors minimized selection bias. Furthermore, the two-week follow-up interval, although relatively short, is commonly adopted in educational intervention studies because it balances short-term retention assessment with reduced participant attrition. Moreover, because participation depended on availability and willingness to enroll, some degree of selection bias may persist despite the use of standardized content and instructors. In addition, participants were aware that they were being evaluated, which may have introduced a Hawthorne effect and temporarily enhanced performance during the practical assessments.

The simulated nature of the practical assessments limits the direct applicability of the results to real clinical settings. Still, simulation provides a safe and controlled environment for skill acquisition and objective performance assessment, which aligns with the study’s educational goals. Finally, the assessment instruments lacked formal validation in Portuguese. However, they were used solely by trained evaluators as structured observation guides, and each station was scored by a single evaluator for all participants, ensuring intra-rater reliability.

Despite these limitations, this study also has important strengths, including standardized training and assessment delivered by the same instructors, prevention of cross-contamination, comparison of groups with distinct clinical profiles, and objective metrics. These features collectively enhance the internal validity of the findings. 

Overall, this study demonstrates that brief, structured FAST training can produce substantial learning gains in both novice physicians and medical students, offering important insights for ultrasound education.

## Conclusions

A brief, structured FAST training program produced substantial gains in theoretical knowledge and practical performance for both physicians and final-year medical students without prior ultrasonography experience. Although physicians scored higher on the post-test assessment, practical performance after training was comparable across groups, suggesting that clinical experience primarily enhances conceptual mastery, while procedural skills can be acquired by novices through targeted, supervised practice. The observed differences in performance patterns, greater technical consistency among physicians, and greater speed among students point to distinct educational needs that could be addressed by tailored instructional strategies.

These findings support the progressive incorporation of ultrasonography into medical training, starting at the undergraduate level and deepening through residency and continuing education. To ensure that early gains translate into safe and durable clinical practice, further studies should evaluate long-term retention and real-world performance. Future research should also examine how training intensity, feedback mechanisms, and supervised clinical exposure interact to produce competence across contexts and learner levels. Such evidence will enable curricula that foster both technical proficiency and sound clinical judgment.
